# World Trade Center Exposure, DNA Methylation Changes, and Cancer: A Review of Current Evidence

**DOI:** 10.3390/epigenomes7040031

**Published:** 2023-12-08

**Authors:** Stephanie Tuminello, Emelie Nguyen, Nedim Durmus, Ramazan Alptekin, Muhammed Yilmaz, Maria Cecilia Crisanti, Matija Snuderl, Yu Chen, Yongzhao Shao, Joan Reibman, Emanuela Taioli, Alan A. Arslan

**Affiliations:** 1 Department of Population Health, NYU Grossman School of Medicine, New York, NY 10016, USA; stephanie.tuminello@nyulangone.org (S.T.);; 2 Institute for Translational Epidemiology, Icahn School of Medicine at Mount Sinai, New York, NY 10016, USA; 3 Department of Medicine, NYU Grossman School of Medicine, New York, NY 10016, USA; 4 Department of Pathology, NYU Grossman School of Medicine, New York, NY 10016, USA; 5 NYU Perlmutter Comprehensive Cancer Center, New York, NY 10016, USA; 6 Division of Environmental Medicine, Department of Medicine, NYU Grossman School of Medicine, New York University, New York, NY 10016, USA; 7 Department of Obstetrics and Gynecology, NYU Grossman School of Medicine, New York, NY 10016, USA

**Keywords:** World Trade Center, epigenetics, DNA methylation, breast cancer, prostate cancer

## Abstract

**Introduction**: Known carcinogens in the dust and fumes from the destruction of the World Trade Center (WTC) towers on 9 November 2001 included metals, asbestos, and organic pollutants, which have been shown to modify epigenetic status. Epigenome-wide association analyses (EWAS) using uniform (Illumina) methodology have identified novel epigenetic profiles of WTC exposure. **Methods**: We reviewed all published data, comparing differentially methylated gene profiles identified in the prior EWAS studies of WTC exposure. This included DNA methylation changes in blood-derived DNA from cases of cancer-free “Survivors” and those with breast cancer, as well as tissue-derived DNA from “Responders” with prostate cancer. Emerging molecular pathways related to the observed DNA methylation changes in WTC-exposed groups were explored and summarized. **Results**: WTC dust exposure appears to be associated with DNA methylation changes across the genome. Notably, WTC dust exposure appears to be associated with increased global DNA methylation; direct dysregulation of cancer genes and pathways, including inflammation and immune system dysregulation; and endocrine system disruption, as well as disruption of cholesterol homeostasis and lipid metabolism. **Conclusion**: WTC dust exposure appears to be associated with biologically meaningful DNA methylation changes, with implications for carcinogenesis and development of other chronic diseases.

## 1. Introduction

The collapse of the Word Trade Center (WTC) buildings in New York City on September 11th, 2001 resulted in acute exposure of rescue workers (Responders) to toxic dust and smoke, as well as local workers, residents, students, and commuters (Survivors) [1]. The dust remained in surrounding areas with potential for continuous resuspension [2,3]. WTC dust, often inches thick, settled in both outdoor and indoor locations [2,4]. Thus, the disaster resulted in chronic as well as acute WTC dust exposure [5], impacting upwards of 400,000 people (both Responders and Survivors). Inhalation of WTC dust resulted in both immediate and long-term adverse health impacts, including epigenetic alterations and cancer development [1,5].

Overall cancer rates of WTC Responders were reported to be 6–14% higher than background rates [2,4,6,7,8,9]. Specifically, excess cases of prostate, thyroid, and skin cancer have been identified among the mostly male Responders [6,8,9,10], and there is evidence that these cancers may in fact be more aggressive compared with those in the general population [11,12,13]. Additionally, there appears to be a dose–response relationship, whereby WTC Responders with prostate cancer with greater levels of WTC dust exposure present with more advanced stages of disease [11]. WTC-associated carcinogens, like heavy metals, have been found in tissues of WTC decedents [14]. Cancers in WTC Survivors, especially cancers in females, remain understudied. Breast cancer is the most common cancer among Survivors [15]; preliminary data suggest that WTC-associated breast cancers are more likely to be poorly differentiated and to have a more aggressive molecular subtype [16]. Given the latency period for cancer development, and the aging population of WTC-exposed individuals, rates of WTC-associated cancer are expected to rise. Therefore, there is an urgent need to better understand the underlying biological relationship between WTC exposure and cancer development.

Carcinogens present in the WTC dust such as metals, asbestos, and organic pollutants, among others [2,4], have previously been described to modify epigenetic status [17,18,19,20,21,22,23,24,25,26]. These modifications include the epigenetic dysregulation of important cancer genes, such as the hypermethylation of tumor suppressor genes *TP53* and *CDKN2A* as a consequence of heavy metal exposure [18,27,28]. Environmental exposure to asbestos, VOCs, PAHs, PCBs, and dioxins, all of which were observed and measured in the WTC dust, have been shown to induce aberrant DNA methylation changes in tumor suppressors and oncogenes, such as *OGG1*, *FOXF1*, *GAS1*, *TP53*, *PTEN*, *BCL3*, and *BRCA1* [26,29,30,31,32,33]. Epigenetic dysregulation may result in undesirable initiation or inhibition of gene expression [34]. Ultimately, cancers can result from a joint accumulation of epigenetic alterations and genetic mutations [34]. Given the chemical makeup of the WTC dust, we [35,36,37] and others [38] have hypothesized that WTC dust exposure results in global, long-term epigenetic alterations capable of promoting tumorigenesis.

We previously described epigenetic profiles from the blood of cancer-free WTC Survivors [35] as well as Survivors with breast cancer [36] and prostate tumor tissue of Responders [37]. In each of these epigenome-wide association studies (EWAS), we found evidence of global and site-specific WTC-associated DNA methylation changes. Another previous EWAS study conducted by Kuan et al. found that both acute and chronic exposure ranking indexes (ERIs) were associated with enriched gene sets involved in cancer, supporting the view that multiple genes play a role in this complex exposure [38]. Our primary objective was to review all the current evidence regarding the association between WTC dust exposure and DNA methylation changes. In addition, we sought to identify emerging biological themes to better understand how WTC-associated DNA methylation may be related to cancer development.

## 2. Methods

We performed a review of available data from the existing studies of exposure assessment and genome-wide DNA methylation status in WTC-exposed populations.

### 2.1. Previous EWAS Studies of WTC Exposure and Cancer

#### 2.1.1. Participants

##### Studies of Cancer-Free Participants

Arslan et al., 2020 [35]: This study was a comparison of global DNA methylation profiles in WTC-exposed Survivors (*n* = 18; average age at blood donation 57.4 years) vs. frequency age-matched cancer-free unexposed females (*n* = 24; average age at blood donation 56.0 years) [35]. WTC-exposed females were enrolled through the WTC Environmental Health Center (WTC EHC) clinic. Funded under the James Zadroga 9/11 Health and Compensation Act, the WTC EHC is a WTC Health Program Center of Excellence for the treatment and surveillance of affected community members (Survivors) [39,40]. Patients at the WTC EHC self-refer into the program and are required by law to have a “certifiable WTC-related condition”, including clinically confirmed cancers, airway and digestive disorders, or mental health conditions [5,39,41]. Participants included in this analysis had a certifiable condition other than cancer, making them cancer-free. The reference group of WTC-unexposed females was selected from the New York University Women’s Health Study (NYUWHS), a prospective cohort recruited to study breast cancer, and included blood samples that had been collected between 1985 and 1991, prior to the WTC disaster. The NYUWHS was previously described in [42,43].

Kuan et al., 2019 [38]: WTC-exposed vs. -unexposed cancer-free males (Responders): Participants were recruited through the Stony Brook WTC Health Program, part of a consortium of Clinical Centers of Excellence in the New York metropolitan area established in 2002 to monitor and treat WTC-related conditions in Responders to the WTC disaster [44]. Male participants were 51.3 years of age on average at the time of blood drawing, predominantly white (83.2%), and nonsmokers (95.7%) [38]. This group identified DNA methylation patterns associated with WTC exposure in the blood of male Responders using an exposure ranking index (ERI) and by comparing high vs. low WTC exposure [38]. A subset of 185 responders with ERI values assessed between February 2012 and March 2014 were included in DNA methylation profiling.

#### 2.1.2. Studies of Cancer Cases

Tuminello et al., 2022 [36]: WTC-exposed vs. -unexposed females with breast cancer (Survivors): To identify an epigenetic profile of WTC-associated breast cancer, the study compared the global DNA methylation profiles in peripheral blood of WTC-exposed (*n* = 28; average age at blood donation 60.4 years) and -unexposed breast cancer cases frequency-matched by age (*n* = 24; average age at blood donation 52.1 years) [36]. WTC-exposed breast cancer cases were enrolled through the WTC EHC clinic for WTC Survivors, whereas the WTC-unexposed pre-diagnostic blood samples of breast cancer cases were selected from the NYU Women’s Health Study (NYUWHS) prospective cohort.

Yu et al., 2022 [37]: WTC-exposed vs. unexposed males with prostate cancer (Responders): To investigate the epigenetic profile of WTC-exposed prostate cancer, the study compared global DNA methylation profiles of WTC-exposed (*n* = 12; average age at cancer diagnosis 54.5 years) and -unexposed (*n* = 13; average age at cancer diagnosis 52.7 years) prostate tumor tissues, frequency-matched by age (±5 years), race/ethnicity (white or other), and Gleason score (scores of 6, 7, or 8). WTC-exposed prostate tumor tissue blocks were obtained through the World Trade Center Tissue Biobank, which contains samples from Responders who participated in the rescue, recovery, and cleanup efforts at the WTC sites enrolled at Mount Sinai in the World Trade Center Health Program (WTCHP) [45]. Funded under the James Zadroga 9/11 Health and Compensation Act of 2010, eligibility criteria for the WTCHP includes type of duties, site location, and dates and hours worked [45]. WTC-unexposed prostate samples were obtained from the Mount Sinai tumor tissue bank.

### 2.2. Sample Collection and Processing

Arslan et al., 2020 [35]: Peripheral blood samples were obtained during routine monitoring visits, with informed consent. WTC-unexposed female blood samples from the NYUWHS were retrieved from storage. Purified DNA from peripheral blood was bisulfite-converted prior to hybridization on the Infinium MethylationEPIC array (Illumina Inc., San Diego, CA, USA) BeadChip [35,46].

Kuan et al., 2019 [38]: All participants provided blood samples for the epigenetic assays. Only English-speaking males were recruited through the Stony Brook WTC-Health Program. Genomic DNA was isolated using standard protocols and used for DNA methylation profiling using the Human Methylation 450K BeadChip (Illumina Inc., San Diego, CA, USA) [38].

Tuminello et al., 2022 [36]: Peripheral blood samples plus informed consent from WTC-exposed breast cancer cases were collected at the WTC EHC. Previously collected blood samples from WTC-unexposed females with pre-diagnostic breast cancer were provided by the NYUWHS prospective cohort. DNA from peripheral blood samples was recovered, purified, and then bisulfite-converted prior to hybridization on the Infinium MethylationEPIC array (Illumina Inc., San Diego, CA, USA) BeadChip [36].

Yu et al., 2022 [37]: WTC-exposed prostate tumor tissue blocks were obtained through the World Trade Center Tissue Biobank [45]. WTC-unexposed prostate samples were obtained from the Mount Sinai tumor tissue bank. Pathologist review and microdissection were performed to ensure that tumor cells comprised more than 80% of formalin-fixed paraffin-embedded (FFPE) tumor tissue samples. DNA was extracted, restored, and bisulfite-treated before being hybridized to the MethylationEPIC array BeadChip (Illumina Inc., San Diego, CA, USA) [37].

### 2.3. Methylation Analysis

Three out of four studies [35,36,37] used the same Infinium Methylation EPIC array (Illumina^®^) BeadChip to determine the DNA methylation status of 866,562 CpG sites [46]. Probes were quantile-normalized, background-adjusted, and adjusted for multiple testing. Probes were annotated using the HumanMethylation850 manifest, as provided by the manufacturer. Detailed methods have been previously reported [35,36,37].

The study by Kuan et al., 2019 used an earlier version of Illumina Infinium Human Methylation 450K BeadChip [38]. DNA methylation data at 485,557 CpG sites were preprocessed and normalized. Statistical significance for CpG association with ERI was assessed by the Wald test, and false-discovery rate (FDR) was used to account for multiple testing. The varying number of CpG sites per gene was accounted for by providing a prior probability for each gene based on gene length, followed by a modified hypergeometric test for overrepresentation of a gene set [38].

### 2.4. Gene Set Enrichment Analysis (GSEA)

Studies by Arslan et al., 2020 and Tuminello et al., 2022 both used the R package Cluster profiler to explore functional pathway enrichment between differentially methylated genes and high-level functional groupings from the Kyoto Encyclopedia of Genes and Genomes (KEGG) pathway map [35,36]. For Yu et al., 2022, differentially methylated gene lists underwent pathway enrichment analysis using the Molecular Signatures Database (MSigDB) hallmark gene set [37]. Because Kuan at al., 2019 did not observe any statistically significant DNA methylation sites after multiple testing adjustment, lists of significant KEGG pathways were used among the top 500 CpG sites [38].

### 2.5. Analysis of Emerging Themes Regarding WTC-Associated DNA Methylation

Next, we explored what common biological themes emerged from three prior EWAS studies of WTC exposure (Arslan et al., 2020; Tuminello et al., 2022; and Yu et al., 2022) [35,36,37]. These studies all included a WTC-unexposed control group and were conducted using the same Infinium MethylationEPIC assay (Illumina) technology. Kuan et al. [38] was not assessed as it did not include a WTC-unexposed group and used an older, more limited Illumina DNA methylation platform. Emerging biological themes were assessed based on the following criteria: consistency in global DNA methylation patterns, GSEA results, and/or DNA methylation of specific genes (based on National Center for Biotechnology Information [NCBI] RefGene annotated names) [47,48] across EWAS studies. Important functionally relevant groups were informed by existing literature: cancer-related [49], immune-related [50], and endocrine-related [51,52,53,54].

## 3. Results

To date, only four studies have been published regarding WTC exposure and genome-wide DNA methylation changes: two studies among cancer-free individuals and two studies among those with cancer (breast and prostate). Two studies each have been conducted on Survivors vs. Responders. Only one study has been conducted using tumor tissue; the rest have been conducted using peripheral blood for DNA extraction. Three of the studies used the same comprehensive Illumina Infinium MethylationEPIC BeadChip for DNA methylation profiling, and all studies conducted some type of GSEA to further explore their results. Statistically significant results were observed in three out of the four studies—notably, in the studies that used a WTC-unexposed group as a control (Table 1).

Evaluation of the EWAS studies [35,36,37] revealed important biological themes enriched in WTC-exposed participants. Specifically, compared with unexposed individuals, WTC-exposed Responders and Survivors appear to have the following:

Theme 1—Increased Global DNA Methylation. Evidence: Independently observed across all EWAS studies (Arslan et al., 2020 [35] (cancer-free WTC Survivors); Tuminello et al., 2022 [36] (Survivors with breast cancer); and Yu et al., 2022 [37] (Responders with prostate cancer)).

Theme 2—Enrichment of Cancer Genes and Pathways. Evidence: GSEA of differentially methylated genes among Arslan et al., 2020 [35] (cancer-free WTC Survivors); Tuminello et al., 2022 [36] (Survivors with breast cancer); and Yu et al., 2022 [37] (Responders with prostate cancer). Differential methylation of tumor suppressors and oncogenes among WTC-exposed cancer cases (breast and prostate).

Theme 3—Inflammation and Immune System Dysregulation. Evidence: GSEA of differentially methylated genes among Arslan et al., 2020 [35] (cancer-free WTC Survivors); Tuminello et al., 2022 [36] (Survivors with breast cancer); and Yu et al., 2022 [37] (Responders with prostate cancer). Dysregulation of immune-related genes among WTC-exposed participants.

Theme 4—Endocrine System Disruption. Evidence: GSEA of differentially methylated genes among Tuminello et al., 2022 [36] (Survivors with breast cancer) and Yu et al., 2022 [37] (Responders with prostate cancer). Dysregulation of endocrine genes among WTC-exposed participants.

Theme 5—Disruption of Cholesterol Homeostasis and Lipid Metabolism. Evidence: GSEA of differentially methylated genes among cancer-free and Survivors with breast cancer [35,36].

More detailed evidence supporting these common biological themes among WTC-exposed participants is presented in Table 2.

## 4. Discussion

It has been hypothesized that WTC fallout exposure is associated with long-term epigenetic consequences, which may play a role in cancer development. Here, we summarize for the first-time evidence from existing EWAS studies of WTC exposure. Although there is a limited number of such studies to date, emerging results suggest that WTC exposure appears to be linked with genome-wide DNA methylation changes that have important biological relevance. This is in keeping with previous work investigating the long-term epigenetic impacts of occupational exposures to dust and fumes, whereby inhalation of small particulate matter induces DNA methylation changes [55,56,57]. A history of exposure to explosive blasts among military members, for example, is encapsulated by DNA methylation profiling of genes linked to commonly reported symptomology such as chronic pain and sleep dysregulation [58].

By evaluating existing WTC EWAS studies concurrently, several important biological themes emerged. Firstly, WTC exposure is associated with a pattern of increased global DNA methylation. This was observed across previous studies of cancer-free WTC Survivors, WTC-exposed Survivors with breast cancer, and WTC-exposed Responders with prostate cancer [35,36,37]. Known or likely roles of DNA hypermethylation in regulation of transcription include silencing gene promoters, silencing gene enhancers, facilitating transcription in gene bodies, and facilitating transcription by maintaining the borders of active promoters or enhancers [59].

Building on this, another emergent theme is that WTC-associated DNA methylation changes are in cancer genes and pathways. The onset of cancer development is known to be accompanied by the activation of oncogenes and/or inactivation of tumor suppressors that are consistent with epigenetic changes to these genes [34,60]. Aberrant DNA methylation at promoters, enhancers, gene bodies, and other regulatory sites of tumor promoters and oncogenes has been shown to be associated with cancer development [59,61]. Statistically significant enrichment of cancer-related pathways was observed across all four EWAS studies in this review. There was also hypermethylation of several important tumor suppressor genes, including high-penetrance (*BRCA1)* and moderate-penetrance (*PALB2)* breast cancer genes [62]. Traditionally, gene hypermethylation is associated with silencing gene expression [59], suggesting that these important tumor suppressor genes could have been “turned off” as a consequence of WTC exposure. *BRCA1* functions primarily in DNA repair and cell cycle control, with *PALB2* being an important gene in the *BRCA* pathway [63]. *BRCA1* and *PALB2* genetic aberrations are associated with both breast and prostate cancer predisposition [62,64]. It remains to be seen, however, what impact DNA methylation of these cancer-related genes may have on cancer survival. Resources like the WTC EHC Pan-Cancer Database will enable us to address this question in future work. As of 1 September 2023, there are 976 prostate cancer and 1213 breast cancer cases documented in the Pan Cancer Database [15,39]. Demographic and treatment data, as well as specific tumor characteristics like Gleason score for prostate cancer, are included in the database, which is periodically linked to the National Death Index [39]. By expanding on these EWAS studies and making use of this rich data source, we will be able to tease apart the effects of WTC-associated DNA methylation on cancer development and survival.

Furthermore, there appears to be WTC-exposure-driven dysregulation of immune and inflammatory responses. Chronic inflammation is associated with an increased risk of malignant disease [65]. Across EWAS studies of WTC exposure and cancer, there is consistent enrichment of immune-related pathways. Moreover, specific and relevant genes appear to be dysregulated. For example, *UBAP2L*, a regulator of multiple important homeostatic pathways including stress-response signaling [66], was observed to be hypermethylated among WTC-exposed breast and prostate cancers. *PRKACA*, which was hypermethylated among WTC-associated cancers, is vital for the attenuation of innate immune response, avoiding host damage during later stages of immune reaction [67]. These results are consistent with previous studies that have demonstrated that WTC exposure is associated with immune system dysregulation [12,68,69]. Gong et al. previously showed that WTC prostate tumors are enriched for proinflammatory Th17 cells [12]. In rats experimentally exposed to WTC dust, gene expression changes in immune–inflammatory response have been observed [12,68,69].

Endocrine disruption is another potential pathway whereby WTC exposure may affect cancer risk [70]. Endocrine-disrupting carcinogens such as pesticides and PCBs were present in the WTC dust [2]. Important endocrine system and cancer-related genes and pathways appear to be dysregulated among WTC-exposed individuals; these include *WRN*, *BRCA1*, *NOTCH1*, *and NTRK1*, among others.

WTC exposure appears to disrupt cholesterol homeostasis and lipid metabolism. Cholesterol metabolism is commonly reprogrammed in cancer cells [71]. Relevant genes and pathways were observed to be dysregulated among WTC-exposed Survivors. Cholesterol dysregulation [12] and increased cardiovascular disease risk [72] have previously been reported among WTC-exposed Responders.

This is the first study to describe and compare studies of epigenetic profiles of both WTC Survivor and Responder populations. However, there are important limitations to the results reported here. First is the relatively small number of EWAS projects published to date, reinforcing the need for future EWAS studies within both WTC Survivor and Responder populations. While EWASs are a powerful tool for identifying biological impacts of exposure, they are prone to several biases, including bias from technical variation or batch effects; inadequate sequencing coverage, especially in instances of extensive PCR amplification; heterogeneity in stability of methylation marks at different CpG sites; variation due to cell type heterogeneity; and bias from unmeasured confounding [73]. There are other limitations specific to each EWAS study that have been described in more detail previously, including a lack of tumor genetic information and germline variation, and limited generalizability to external populations due to selection bias [35,36,37,38]. Notably, in the study of WTC-associated breast cancer, blood from unexposed females came from pre-diagnostic breast cancer cases [36]. Because DNA methylation alterations are stable during long-term sample cryopreservation [74,75], it is assumed that the observed DNA methylation changes existed prior to breast cancer development. Comparability of the EWAS studies is also limited by several factors. First, the EWAS studies used for these analyses varied in terms of participant sex (male and female) and sample type (blood or tissue)—variables that themselves likely impact epigenetic status [73]. While the three reviewed EWAS studies all used the same Illumina technology for DNA methylation profiling, important data processing steps differed between them, including steps related to normalization and adjustment for cell types. Methylation effects could also differ depending on the specific CpG location [26,59,73]. A better understanding of WTC cancer-development will be aided by additional larger EWAS studies of WTC exposure among cancer-free Survivors and Responders, as well as WTC-exposed persons with various cancer types.

## 5. Conclusions

There is an urgent need to better understand mechanisms of WTC-related cancers. Assessing previously published studies revealed a potential relationship between WTC exposure and epigenetic dysregulation, with relevance for functional consequences and cancer risk. Additional EWAS studies are needed to better understand the interplay between WTC exposure, epigenetic dysregulation, and cancer development. The insights reported here will hopefully be validated and expanded upon in future work.

## Figures and Tables

**Table 1:** Description of previous EWAS.

	EWAS of WTC Exposure and DNA Methylation Changes		EWAS of WTC Exposure and DNA Methylation Changes among Cancer Cases	
	Kuan et al., 2019, Eur. J. Cancer Prev. [38]	Arslan et al., 2020, Int. J. Environ. Res. Public Health [35]	Tuminello et al., 2022, Int. J. Environ. Res. Public Health [36]	Yu et al., 2022, Carcinogenesis [37]
WTC Group	Responders	Survivors	Survivors	Responders
Sample Type	Blood	Blood	Blood	Tumor Tissue
Sex	Male	Female	Female	Male
Cases	Male Responders with high WTC exposure ranking index (ERI) (*n* = 116)	WTC-exposed cancer-free females (*n* = 18)	WTC-exposed females with breast cancer (*n* = 28)	WTC-exposed males with prostate cancer (*n* = 13)
Controls	Male Responders with low WTC ERI (*n* = 69)	WTC-unexposed cancer-free females (*n* = 24)	WTC-unexposed females with pre-diagnostic breast cancer (*n* = 24)	WTC-unexposed males with prostate cancer (*n* = 15)
Matching	Not matched; similar in terms of race and smoking history	Frequency-matched on age	Frequency-matched on age	Frequency-matched on age, race/ethnicity, and Gleason score
DNA Methylation Platform	Human Methylation 450K BeadChip (Illumina^®^) 485,557 CpG sites	Infinium Methylation EPIC array (Illumina^®^) 866,562 CpG sites	Infinium Methylation EPIC array (Illumina^®^) 866,562 CpG sites	Infinium Methylation EPIC array (Illumina^®^) 866,562 CpG sites
Main Study Findings	Several cancer-related pathways were enriched in participants with high WTC ERI	Increased global methylation among WTC-exposed; statistically significant CpG sites linked to DMGs associated with WTC exposure; several pathways were enriched in the WTC-exposed participants	Increased global methylation among WTC-exposed; statistically significant CpG sites linked to DMGs associated with WTC exposure and breast cancer; several pathways were enriched in the WTC-exposed participants	Increased global methylation among WTC-exposed; statistically significant CpG sites linked to DMGs associated with WTC exposure and prostate cancer; several pathways were enriched in the WTC-exposed participants

**Table 2:** WTC-associated DNA Methylation: Emerging Biological Themes.

Theme	Supporting Evidence
Theme 1—Increased Global DNA Methylation	All EWAS studies (Arslan et al., 2020 (cancer-free WTC Survivors) [35]; Tuminello et al., 2022 (Survivors with breast cancer) [36]; and Yu et al., 2022 (Responders with prostate cancer) [37]) independently observed increased global DNA methylation associated with WTC exposure.
Theme 2—Dysregulation of Cancer Genes and Pathways	Pathways enrichment showed dysregulation of genes in important cancer-related functional pathways, notably for cell adhesion and mobility, even among WTC cancer-free Survivors. Arslan et al., 2020 (cancer-free WTC Survivors) [35]: demonstrated enrichment in Endocytosis, MAPK Signaling, Cell Cycle, Viral Carcinogenesis, mTOR Signaling, Base Excision Repair, and Regulation of the Actin Cytoskeleton and Axon Guidance pathways, among others. Tuminello et al., 2022 (Survivors with breast cancer) [36]: observed pathway enrichment of Endocytosis, Viral Carcinogenesis, Non-small-Cell Lung Cancer, ErbB Signaling, Adherens Junction, Regulation of the Actin Cytoskeleton, and Focal Adhesion pathways, among others. Yu et al., 2022 (Responders with prostate cancer) [37]: observed Epithelial Mesenchymal Transition, Hypoxia, MYC Targets, and Apical Junction and Miotic Spinal pathway enrichment, among others. Among both studies of WTC-associated cancers (Tuminello et al., 2022 (Survivors with breast cancer) [36] and Yu et al., 2022 (Responders with prostate cancer) [37]), increased DNA methylation was observed for the *BRCA1*, *NOTCH1*, *PALB2*, and *WRN* tumor suppressor genes. Oncogene dysregulation was also observed, including of *KRAS and NTRK1*.
Theme 3—Inflammation and Immune System Dysregulation	Immune-related pathways were enriched among EWAS studies of WTC exposure: Arslan at al., 2020 (cancer-free WTC Survivors) [35]): Vibrio Cholerae Infection pathway. Tuminello et al., 2022 (Survivors with breast cancer) [36]: Human Papillomavirus Infection, Human Cytomegalovirus Infection, Human Immunodeficiency Virus 1, Bacterial Invasion of Epithelial Cells, T Cell Receptor Signaling, B Cell Receptor Signaling, and Inflammatory Mediator of TRP Channels pathways. Yu et al., 2022 (Responders with prostate cancer) [37]: TNFA Signaling via NFKB and complement pathways. The following immune-related genes were observed to be dysregulated in multiple EWAS studies of WTC exposure and cancer (Tuminello et al., 2022 (Survivors with breast cancer) [36] and Yu et al., 2022 (Responders with prostate cancer) [37]): *TRAF1*, *TUBG1*, *GFPT1*, *PIP5K1C*, *ANGEL1*, *AP2B1*, *UNC5B*, *PLXND1*, *UBAP2L*, *PRKACA*, and *SERPINF1.*
Theme 4—Endocrine System Disruption	Preliminary evidence suggests that disruption of endocrine pathways plays a role in WTC-associated carcinogenesis: Tuminello et al., 2022 (Survivors with breast cancer) [36]: Thyroid Hormone Signaling; Parathyroid Hormone Synthesis, Secretion, and Action; and Aldosterone Synthesis and Secretion pathways. Yu et al., 2022 (Responders with prostate cancer) [37]: and Androgen Response pathway enrichment. Genes *DOK1*, *WRN*, *PDE4A*, *BRCA1*, *DCUN1D1*, *NTRK1*, *GRK5*, *KIDINS220*, *NOTCH1*, and *FLNC* are all implicated in endocrine systems but appear to be dysregulated among WTC-associated cancers (Tuminello et al., 2022 (Survivors with breast cancer) [36] and Yu et al., 2022 (Responders with prostate cancer) [37]).
Theme 5—Disruption of Cholesterol Homeostasis and Lipid Metabolism	Cholesterol is an important precursor for cell membrane creation and maintenance; however, evidence suggests that cholesterol homeostasis is disturbed among WTC-exposed individuals, as is lipid metabolism. WTC-associated DNA methylation changes have been observed to be enriched in relevant pathways, including the following: Arslan et al., 2020 (cancer-free WTC Survivors) [35]: Glycerophospholipid Metabolism and Inositol Phosphate Metabolism pathways. The Phosphatidylinositol Signaling System pathways were dysregulated in multiple studies (Arslan et al., 2020 (cancer-free WTC Survivors) [35] and Tuminello et al., 2022 (Survivors with breast cancer) [36]).

## Data Availability

No new data were created or analyzed in this study. Data sharing is not applicable to this article.
